# Engagement Enhancement Based on Human-in-the-Loop Optimization for Neural Rehabilitation

**DOI:** 10.3389/fnbot.2020.596019

**Published:** 2020-11-12

**Authors:** Jiaxing Wang, Weiqun Wang, Shixin Ren, Weiguo Shi, Zeng-Guang Hou

**Affiliations:** ^1^School of Artificial Intelligence, University of Chinese Academy of Sciences, Beijing, China; ^2^State Key Laboratory of Management and Control for Complex Systems, Institute of Automation, Chinese Academy of Sciences, Beijing, China; ^3^Chinese Academy of Sciences Center for Excellence in Brain Science and Intelligence Technology, Beijing, China

**Keywords:** human-in-the-loop optimization, EEG based neural engagement, sEMG based muscle activation, tracking accuracy, neural rehabilitation

## Abstract

Enhancing patients' engagement is of great benefit for neural rehabilitation. However, physiological and neurological differences among individuals can cause divergent responses to the same task, and the responses can further change considerably during training; both of these factors make engagement enhancement a challenge. This challenge can be overcome by training task optimization based on subjects' responses. To this end, an engagement enhancement method based on human-in-the-loop optimization is proposed in this paper. Firstly, an interactive speed-tracking riding game is designed as the training task in which four reference speed curves (RSCs) are designed to construct the reference trajectory in each generation. Each RSC is modeled using a piecewise function, which is determined by the starting velocity, transient time, and end velocity. Based on the parameterized model, the difficulty of the training task, which is a key factor affecting the engagement, can be optimized. Then, the objective function is designed with consideration to the tracking accuracy and the surface electromyogram (sEMG)-based muscle activation, and the physical and physiological responses of the subjects can consequently be evaluated simultaneously. Moreover, a covariance matrix adaption evolution strategy, which is relatively tolerant of both measurement noises and human adaptation, is used to generate the optimal parameters of the RSCs periodically. By optimization of the RSCs persistently, the objective function can be maximized, and the subjects' engagement can be enhanced. Finally, the performance of the proposed method is demonstrated by the validation and comparison experiments. The results show that both subjects' sEMG-based motor engagement and electroencephalography based neural engagement can be improved significantly and maintained at a high level.

## 1. Introduction

One of the most common sequela following stroke or cerebral injury is motor dysfunction, which seriously affects a person's quality of life. To regain their motor abilities, patients need to perform significant repetitive physical therapy, which is prone to boredom and often leads to low engagement. Previous studies have demonstrated that high levels of motivation and engagement are essential for obtaining relatively satisfactory rehabilitation outcomes (Tupper and Henley, 1987; Grant et al., 2004; Holden, 2005; Colombo et al., 2007). Developing a rehabilitation training method that can be used to reduce the boredom of the training tasks and promote engagement of the patients is therefore essential for post-stroke rehabilitation.

Engagement can be defined as a complex construct, which is driven by motivation and executed through active participation (Li et al., 2016). It was reported that motivating and empowering patients by providing them with the perception of control can improve patients' engagement, thus expediting the achievement of the patient's rehabilitation goals (Lenze et al., 2004; Dunn and Dougherty, 2005). Positive feedback can promote patient morale and engagement (Paolucci et al., 2012). Virtual reality (VR), which can be used to provide the task-specific training and intuitive multi-sensory feedbacks, has been therefore been widely applied in post-stroke rehabilitation.

The adaptive adjustment of the training task is often used for improving patient engagement. The challenge level of training tasks, which is one of the main sub-factors that contribute to engagement, can be adjusted to match a patients' motor abilities by use of training task adaptation (Csikszentmihalyi and Csikzentmihaly, 1990; Yannakakis and Hallam, 2009; Xu et al., 2017, 2018; Agarwal and Deshpande, 2019). In 2003, Krebs et al. proposed a performance-based progressive robotic therapy method (Krebs et al., 2003). In the Krebs's method, patients' active forces and motion-accuracy-based performance were used to customize the stiffness parameters of the robot controller and thus to maximize the recovery benefits (Krebs et al., 2003). Similarly, in 2014, an intelligent game engine was specifically designed for post-stroke rehabilitation, where the game parameters can be adjusted in real time according to patients' performance based on a Bayesian framework (Pirovano et al., 2014). Besides, interaction forces, muscle activity, or other physical or physiological parameters also have been used for training challenge adaption (Krebs et al., 2003; Novak et al., 2011; Luo et al., 2019).

However, due to the complexity of the training tasks and human-machine systems, the adaptive task adjustment-based engagement enhancement methods can hardly find an optimal design of the training tasks. This can be obtained via the optimization method, though this has rarely been studied. Besides, considering that physiological and neurological differences among individuals can cause divergent responses to the same task, and the responses can further change considerably during the training (Gordon and Ferris, 2007; Zelik et al., 2011; Jackson and Collins, 2015; Selinger et al., 2015; Quesada et al., 2016), subjects' physiological variations or responses also need to be considered during the training task optimization. Subjects' responses based training task optimization belongs to human-in-the-loop optimization (HILO).

To the best of our knowledge, HILO method-based training task optimization has rarely been studied. All the key steps of the HILO, including the training task modeling and design of the objective function and the optimization algorithm, can affect the optimization results. On one hand, the parameters used for modeling the training task should be sensitive to the engagement variation, based on which subjects' engagement can be improved through the parameter optimization. On the other hand, adding human responses to the engagement enhancement optimization loop also makes the optimization difficult to implement due to the time-varying dynamics of the subjects, such as the self-adaptation ability, the strong history dependence, and other complex neurocognitive factors (Gordon and Ferris, 2007; Selinger et al., 2015). Both the objective function and the optimization algorithm should therefore be insensitive to human dynamic variation and noises.

In this paper, an HILO-based engagement enhancement method is proposed. The original contributions of this study can be summarized as follows: ① an optimization-based engagement enhancement method is proposed, ② and the proposed HILO method is tolerant of both measurement noises and human adaptation.

Firstly, an interactive speed-tracking riding game is designed as the training task. In the task, subjects are asked to track the reference trajectory, which is constructed by four reference speed curves (RSCs), as accurately as possible. Each RSC is modeled using a piecewise function and determined by the starting velocity, transient time, and end velocity. By parameterizing the RSC, it is possible to optimize the difficulty of the training task, which is a key factor affecting a user's engagement level.

Then, the objective function is designed by consideration of the tracking accuracy (TA) and the muscle activation (MA), based on which subjects' physical and physiological responses can be evaluated simultaneously. By maximizing the subject's TA and MA concurrently, the difficulty of the training task can be optimized to match subject's current motor ability and physiological state.

Moreover, the covariance matrix adaptation evolution strategy (CMA-ES) is used to optimize the parameters of the RSCs (Hansen, 2006; Akimoto et al., 2012; Zhang et al., 2017; Maki et al., 2020). In the CMA-ES, neither objective function values nor their derivatives are used directly, and each generation is evaluated independently. It is therefore relatively tolerant of both measurement noises and human adaptation. By optimization of the RSCs persistently, the objective function can be maximized and subject engagement enhanced.

Finally, the performance of the proposed HILO method is demonstrated through a comparison experiment. The results show that both TA and MA can be improved significantly. Moreover, the subjects' neural engagement can also be improved significantly and maintained at a high level.

## 2. Task Modeling and Optimization

An HILO method is designed to enhance the subjects' engagement in this study. Details of the HILO method are given in the following text.

### 2.1. Modeling the Training Task

**Figure 1:**
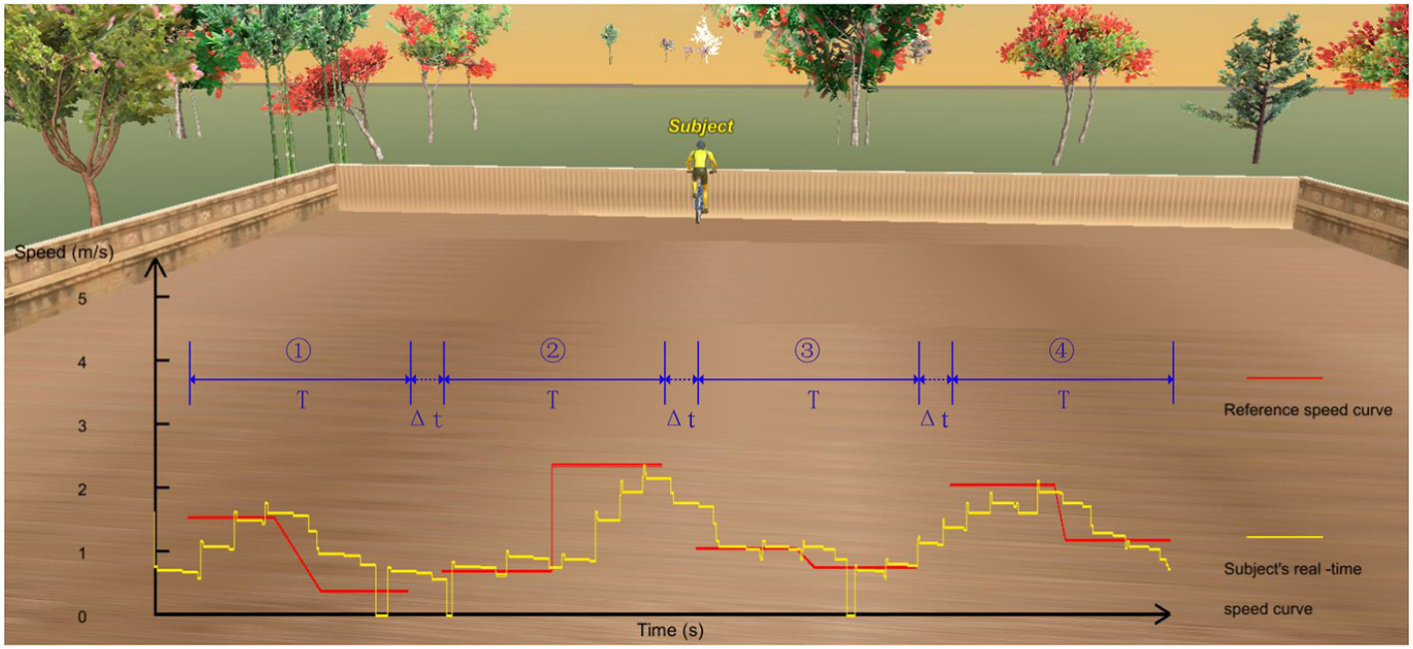
The virtual scene of the designed speed-tracking riding game. The four red lines represent the four RSCs, which are used to construct the reference trajectory in each generation, and the yellow line represents subject' actual speed tracking trajectory.

Based on the previous study (Wang et al., 2019), an interactive speed-tracking riding game is designed as the training task, which can be seen from Figure 1. During the training, subjects need to try their best to track the reference trajectory.

To increase the complexity of the task and, meanwhile, facilitate optimization, four relatively simple RSCs were used to construct the reference trajectory in each generation, which can be seen from Figure 1. The *T* and △*t* are the period of each RSC and the time interval between the two adjacent RSCs, respectively. In this study, the *T* and △*t* were set to 12 and 3 s, respectively. △*t* is designed to give the subjects enough time to adjust their riding speeds to better complete the subsequent tracking task.

**Figure 2:**
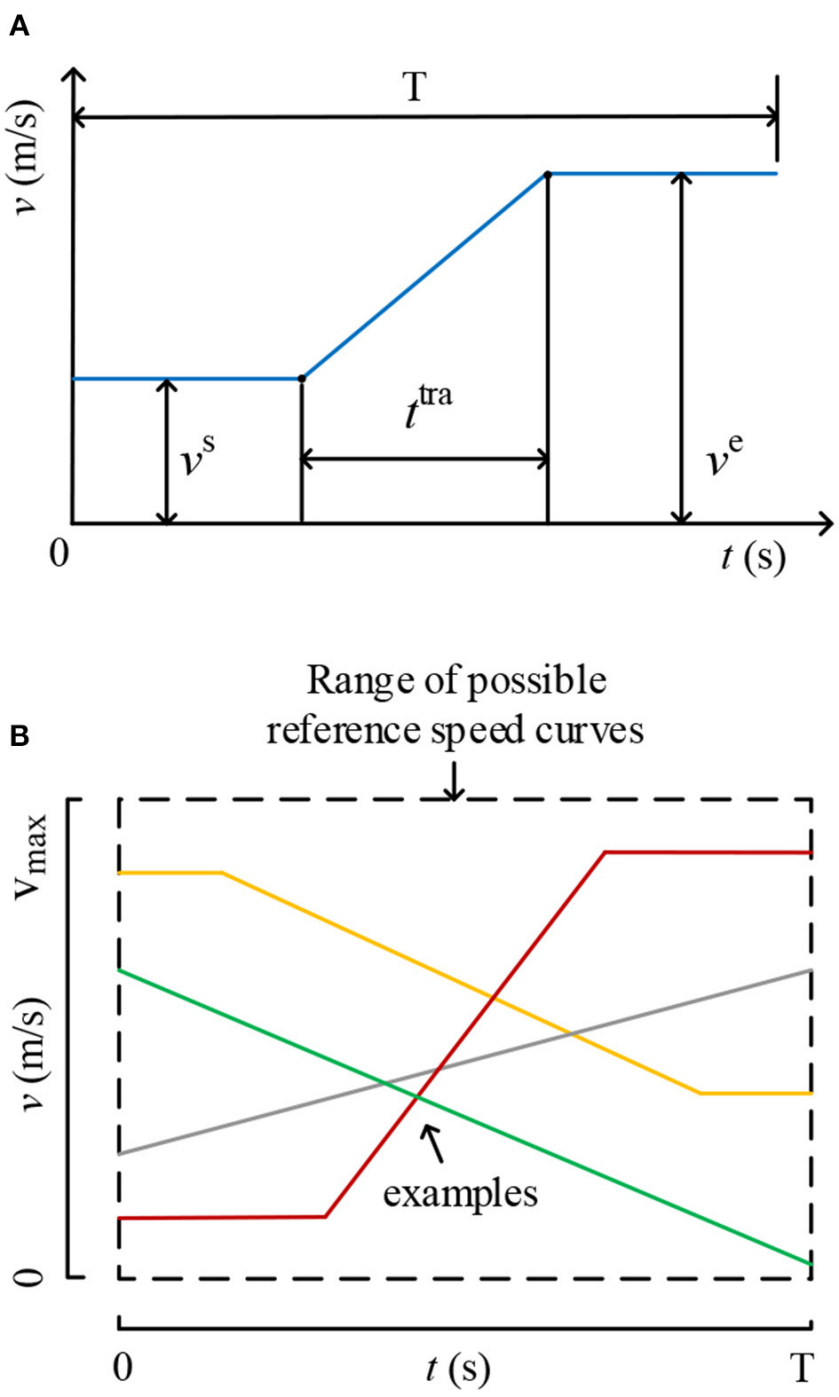
**(A)** Parameterization of each RSC. **(B)** Examples of possible RSCs.

Specifically, each RSC is determined by three parameters: starting velocity (*v*^s^), transient time (*t*^tra^), and end velocity (*v*^e^). The definition of these three parameters is given in Figure 2. It can be seen that each RSC can be defined as a piecewise function of time, which is given by the following:

(1) $$\begin{array}{l} V^{\text{ref}}(t)=\{\begin{array}{ll} v^{\text{s}} & \;t\in [0,\frac{\text{T}-t^{\text{tra}}}{2}]\\ v^{\text{s}}+\frac{v^{\text{e}}-v^{\text{s}}}{t^{\text{tra}}}(t-t^{\text{tra}}) & t\in (\frac{\text{T}-t^{\text{tra}}}{2},\frac{\text{T}+t^{\text{tra}}}{2})\\ v^{\text{e}} & \;t\in [\frac{\text{T}+t^{\text{tra}}}{2},\text{T}] \end{array} \end{array}$$

Based on the parameterized model, the difficulty of the training task, which is a key factor affecting the engagement, can be optimized. A wide range of possible RSCs can be obtained by Equation (1), and some examples of possible RSCs are given in Figure 2B.

In this study, constraints given in Equation (2) are used to avoid appearance of some weird RSCs, such as too high reference speeds and sharp change of the speed.

(2) $$\begin{array}{l} 0\leq v^{\text{s}}\leq 6,\;0\leq v^{\text{e}}\leq 6,\\ \;t^{\text{tra}}\geq \frac{\left|v^{\text{e}}-v^{\text{s}}\right|}{6} \end{array}$$

where, the units of *v*^s^ (*v*^e^) and *t*^tra^ are meters per second (m/s) and seconds (s), respectively.

### 2.2. Design of the Objective Function

Both electroencephalography (EEG) and surface electromyogram (sEMG)-based physiological responses, which can reflect subjects' engagement levels during the training, can be used to construct the objective function (Zimmerli et al., 2013; Tacchino et al., 2016). Compared with sEMG, the EEG signals are much weaker (microvolt level), and they can be easily contaminated by the environment noises or the subjects' physiological variation, such as emotional fluctuation. If the EEG based objective function is used for the HILO, the parameters to be optimized can hardly converge to the optima. In this paper, sEMG-based MA is thus chosen to measure subjects' physiological response. Besides, the subjects' physical response is evaluated by TA. By maximizing subject's TA and MA concurrently, the difficulty of the training task can be optimized to match the subject's current motor ability and physiological state. On one hand, a relatively high TA can be obtained when the tracking task is designed relatively easily. However, speed-tracking tasks that are too easy can easily lead to a phenomenon where a subject's MA is relatively low, which is not beneficial for the restoration of muscle strength. On the other hand, a relatively high MA can be obtained when the tracking task is designed relatively difficult. Tasks that are too difficult, however, can cause the subjects to become discouraged and unwilling to continue the training. Simultaneously maximizing TA and MA can result in a suitable challenging task for a specific subject, thus enhancing the subjects' engagement. In this paper, TA and MA are therefore used to construct the objective function.

Specifically, the TA is given by the following:

(3) $$\begin{array}{l} F_{i}^{\text{TA}}=-\frac{||V_{i}^{\text{ref}}-V_{i}^{\text{act}}|{|}_{2}}{\sqrt{\text{N}}},\;i=1,2,3,4 \end{array}$$

where, ||*||_2_ means the calculation of the L2-norm. $V_{i}^{\text{ref}}\in {\mathbb{R}}^{\text{N}}$ and $V_{i}^{\text{act}}\in {\mathbb{R}}^{\text{N}}$ are the reference speed vector and subject's actual speed vector with 100 Hz sample rate acquired during tracking the *i*th RSC in each generation. The period of each RSC is 12 s, therefore, *N* is equal to 1,200. In this study, the subject's actual speeds are collected using a data acquisition card and transmitted to the computer via TCP/IP protocol.

As for the sEMG-based MA, it has been proved that, when subjects are focused on the training, the root mean square (RMS) of sEMG signals can become bigger (Zimmerli et al., 2013). In this paper, RMS is consequently used to indicate subjects' MA.

(4) $$\begin{array}{l} F_{i}^{\text{MA}}=\frac{||S_{i}^{\text{EMG}}|{|}_{2}}{\sqrt{\text{M}}},\;i=1,2,3,4 \end{array}$$

where, $S_{i}^{\text{EMG}}\in {\mathbb{R}}^{\text{M}}$ represents the amplitude vector of the acquired sEMG signals with 400 Hz sample rate acquired during tracking the *i*th RSC in each generation, and *M* is equal to 4,800.

The muscles contributing to cycling motion, including rectus femoris (RF), hamstring, soleus, and gastrocnemius, are mainly considered. During the pre-experiment, it was found that the RF muscle had the highest activation during the cycling training, and it is therefore used to calculate the MA in this study. Delsys Trigno^TM^ device with a 1111.11 Hz sample rate was used to monitor muscle activities during cycling. The raw sEMG signals were first filtered by a band-pass butterworth filter (10–400 Hz) and a notch filter (50 Hz) to reduce the effects of noise and power line interference. Then, the subjects' average MA can be calculated using Equation (4).

Finally, the objective function, which is equal to the weight sum of the TA and MA, can be given as follows:

(5) $$\begin{array}{l} F_{i}^{\text{OBJ}}=F_{i}^{\text{TA}}+\alpha F_{i}^{\text{MA}},\;i=1,2,3,4 \end{array}$$

where, $F_{i}^{\text{TA}}$ and $F_{i}^{\text{MA}}$ represent the values of TA and MA of the *i*th sub-racking task in each generation, respectively. α is a scaling coefficient to weight $F_{i}^{\text{TA}}$ and $F_{i}^{\text{MA}}$, and it is set to 1 in this study.

### 2.3. CMA-ES Based HILO

In this study, the optimization problem for engagement enhancement can be defined as follows.

Parameters to be optimized are the following:

(6) $$\begin{array}{l} m_{i}=\left[v_{i}^{\text{s}},\;t_{i}^{\text{tra}},\;v_{i}^{\text{e}}\right],\;i=1,2,3,4 \end{array}$$

The objective function to be maximized is the following:

(7) $$\begin{array}{l} F_{i}^{\text{OBJ}}=F_{i}^{\text{TA}}+F_{i}^{\text{MA}},\;i=1,2,3,4 \end{array}$$

Constraints to be satisfied are the following:

(8) $$\begin{array}{l} 0\leq v_{i}^{\text{s}}\leq 6,\;0\leq v_{i}^{\text{e}}\leq 6,\\ t_{i}^{\text{tra}}\geq \frac{\left|v^{\text{e}}-v^{\text{s}}\right|}{6},\;i=1,2,3,4 \end{array}$$

It can be seen that the optimization problem of this paper is strongly non-linear, and it can be easily disturbed by the time-varying dynamics of the subjects. Therefore, CMA-ES, which is relatively tolerant of both measurement noises and human adaptation, is applied to optimize the training task in this paper. No gradient calculation is involved in the CMA-ES, which makes this method robust and feasible even for a non-continuous problem. With each iteration, new task-setting parameters are generated stochastically using a multivariate normal distribution, and the distribution parameters, including the mean vector, the covariance matrix, and the evolution paths, are updated with successful candidate solutions and their objective value ranking. In this paper, the algorithm of the CMA-ES (Hansen, 2006; Maki et al., 2020) based HILO is given in Algorithm 1.

**Algorithm 1:** CMA-ES based HILO.

1: *n*: number of parameters to be optimized, defaults to 3.
2: $\bar{m}\in {\mathbb{R}}^{n}$, mean vector, initialized with [2.5, 4, 5.5].
3: ***p*_*c*_**, $p_{\sigma }\in {\mathbb{R}}^{n}$, evolution paths, initialized with 0.
4: σ: step size, initialized with 2.
5: ***C*** ∈ ℝ^*n*×*n*^: covariance matrix, initialized with ***I***.
6: λ: population size of each generation, defaults to 4.
7: λ^opt^: number of candidate population, defaults to 2.
8: $w\in {\mathbb{R}}^{\lambda^{\text{opt}}},\mu_{w}$: weight constants for $\bar{m}$, σ and ***C*** update.
$w\left(i\right)=\frac{log\left(\lambda^{\text{opt}}+1/2\right)-log\left(i\right)}{\overset{\lambda^{\text{opt}}}{\underset{i=1}{\sum }}\left(log\left(\lambda^{\text{opt}}+1/2\right)-log\left(i\right)\right)},i=1,..,\lambda^{\text{opt}}$.
$\mu_{w}=1/\overset{\lambda }{\underset{i=1}{\sum }}w{\left(i\right)}^{2}$.
9: *μ*_*n*_: approximated norm of the expected value of n-dimension normal distribution.
$\mu_{n}=n^{1/2}\left(1-1/4n+1/21n^{2}\right)$.
10: *c*_*c*_, *c*_σ_: cumulation factors for evolution.
*c*_*c*_ = (4 + μ_*w*_/*n*)/(*n* + 4 + 2μ_*w*_/*n*).
*c*_σ_ = (μ_*w*_ + 2)/(*n* + μ_*w*_ + 5).
11: *d*_σ_: damping factor for σ update.
*d*_σ_ = 1 + 2*max*(0, *sqrt*((μ_*w*_ − 1)/(*n* + 1)) − 1) + *c*_σ_.
12: *c*_1_, *c*_μ_: learning rate for covariance update.
$c_{1}=2/\left({\left(n+1.3\right)}^{2}+\mu_{w}\right)$.
$c_{\mu }=min\left(1-c_{1},2\left(\mu_{w}-2+1/\mu_{w}\right)/\left({\left(n+2\right)}^{2}+\mu_{w}\right)\right)$.
13: **for** each generation **do**
14: **for** *i* = 1 → λ **do**
15: $m_{i}=\bar{m}+\sigma N\left(0,C\right)$.
16: Generate the RSC and $V_{i}^{\text{ref}}$ with ***m***_*i*_ using Equation (1).
17: **end for**
18: Tracking, acquire $V_{i}^{\text{act}}$ and $S_{i}^{EMG}$, where *i* = 1, ..., λ.
19: **for** *i* = 1 → λ **do**
20: Compute $F_{i}^{\text{TA}}$ with $V_{i}^{\text{ref}}$ and $V_{i}^{\text{act}}$ using Equation (3).
21: Compute $F_{i}^{\text{MA}}$ with $S_{i}^{\text{EMG}}$ using Equation (4).
22: Compute $F_{i}^{\text{OBJ}}$ with $F_{i}^{\text{TA}}$ and $F_{i}^{\text{MA}}$ using Equation (7).
23: **end for**
24: Get *I*^opt^: the indices of the top λ^opt^ values of *F*^OBJ^
in descending order.
25: $m^{\text{opt}}={\left\{m_{i}\right\}}_{i\in I^{\text{opt}}}$.
26: $m_{d}=\left(\underset{i=1\to \lambda^{\text{opt}}}{\sum }\left(m^{\text{opt}}\left(i\right)\right)w\left(i\right)-\bar{m}\right)/\sigma$
27: Update $p_{\sigma }=\left(1-c_{\sigma }\right)p_{\sigma }+{\left(c_{\sigma }\left(2-c_{\sigma }\right)\mu_{w}\right)}^{1/2}\frac{1}{\sqrt{C}}m_{d}$
28: Update $p_{c}=\left(1-c_{\sigma }\right)p_{c}+{\left(c_{c}\left(2-c_{\sigma }\right)\mu_{w}\right)}^{1/2}m_{d}$
29: Update $\bar{m}=\underset{i=1\to \lambda^{\text{opt}}}{\sum }\left(m^{\text{opt}}\left(i\right)\right)w\left(i\right)$.
30: Update $\sigma =\sigma \exp\left[\frac{c_{\sigma }}{d_{\sigma }}\left(\frac{\left||p_{\sigma }|\right|}{\mu_{w}}-1\right)\right]$
31: Update $C=C+c_{1}\left(p_{c}{p_{c}}^{T}-C\right)+c_{\mu }\left({\sigma^{2}m_{d}}^{2}-C\right)$
32: **end for**

In each generation, four groups of the RSC parameter settings, (***m***_*i*_)_*i*=1,2,3,4_, are generated stochastically using a multivariate normal distribution $N\left(\bar{m},\sigma^{2}C\right)$, to form the tracking trajectory of the current generation.

(9) $$\begin{array}{l} m_{i}=\bar{m}+\sigma N\left(0,C\right),\;i=1,2,3,4 \end{array}$$

where, $\bar{m}=\left[\bar{v^{\text{s}}},\bar{t^{\text{tra}}},\bar{v^{\text{e}}}\right]$. Specifically, $\bar{m}$ is the mean vector of the parameters to be optimized, and it determines the search space of the ***m***_*i*_. σ is the step parameter, which determines the size and intensity of the search range. ***C*** is the covariance matrix, which determines the shape of the distribution. In this study, $\bar{m}$, σ and ***C*** are initialized with [2.5, 4, 5.5], 2 and ***I***, respectively.

When the tracking task in each generation is finished, the average TA and MA can be calculated according to the subjects' responses. The value of the objective function can consequently be calculated by Equation (7). Then, according to the value ranking of ${\left(F_{i}^{\text{OBJ}}\right)}_{i=1,2,3,4}$, λ^opt^ parameter settings, ***m***^opt^, can be obtained, and these are used to update the two evolution paths, ***p***_σ_ and ***p***_*c*_. Finally, based on the ***m***^opt^, ***p***_σ_, and ***p***_*c*_, $\bar{m}$, σ, and ***C***, which are used to generate the tracking trajectory of the next generation, can also be updated. The tracking trajectory in each generation can therefore be updated continuously by using the current multivariate normal distribution $N\left(\bar{m},\sigma^{2}C\right)$. It can be seen that, by using the proposed CMA-ES based HILO method, the training task can be optimized automatically and constantly to achieve engagement enhancement.

### 2.4. Neural Engagement Evaluation Method

Since the purpose of the proposed optimization method is to enhance and maintain subjects' engagement during the rehabilitation training, the subjects' neural engagement levels were also evaluated in this study.

Neural engagement, which is an essential factor in promoting neural reorganization and compensation, is considered to be proportional to the level of concentration (attention) during the rehabilitation training (Park et al., 2014; Li et al., 2016). Previous researches have demonstrated that EEG signals in the theta and beta bands can be used to quantitatively represent subjects' attention states (Mann et al., 1992; Harmony et al., 1996). Good performance and high attention level have been proven to be related to the decrease of the theta rhythm power and the increase of the beta rhythm power (Kropotov, 2009; Gürkök et al., 2011; Arns et al., 2012; Loo and Makeig, 2012; Marshall et al., 2013). The EEG-based theta to beta power ratio (TBR) was thus used to measure subjects' neural engagement, which can be given by the following:

(10) $$\begin{array}{l} TBR=-\frac{E\left(\text{theta}\right)}{E\left(\text{beta}\right)}\\ E_{\text{n}}=\frac{\sum_{i=1}^{5}TBR\left(i\right)}{5} \end{array}$$

where *E*(theta) and *E*(beta) represent the energy of theta and beta bands in the latest 3 s, respectively. *TBR* was calculated every 3 s. *E*_n_, which is equal to the mean of the latest 5 *TBR* values, was used to indicate subjects' attention and neural engagement. A high *E*_n_ represents a high level of neural engagement.

**Figure 3:**
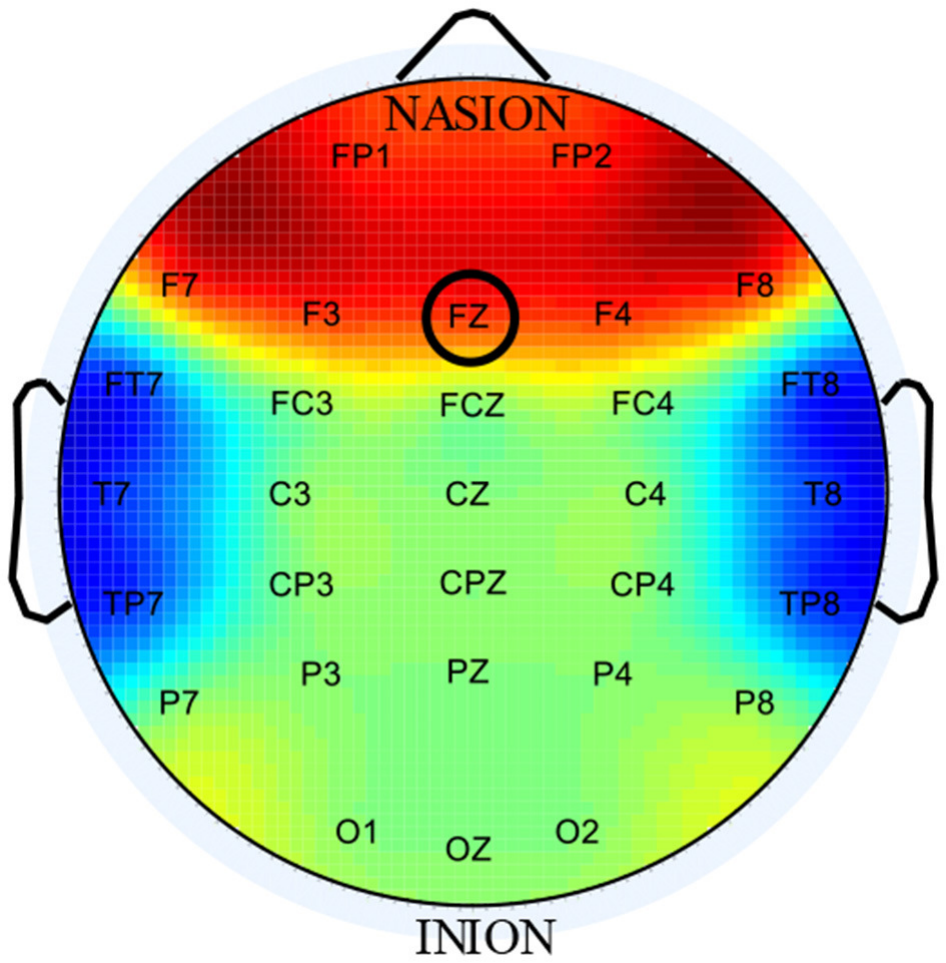
International 10–20 system. The red and blue areas represent the frontal and temporal regions, respectively.

By considering that EEG activities in the frontal and temporal lobes are most related to human engagement levels (Barkley et al., 1992; Mann et al., 1992), EEG signals acquired from these two brain regions can be used to compute *E*_*n*_, which can be seen from Figure 3. However, EEG signals, especially collected during cycling, can be easily contaminated by ocular artifacts (OAs) and EMG (Frølich et al., 2015; Kline et al., 2015; Zink et al., 2016). Many studies focused on eliminating the artifacts have been conducted, but the results are still not satisfying.

For the term of OAs, blinking or moving the eyes can produce large electrical potential, which will spread across scalp and contaminate the EEG signals. EEG signals in the forehead (FP1 and FP2) are most susceptible to OAs (Babu and Prasad, 2011). For the term of EMG artifacts, subject movement (riding) can introduce some muscle artifacts to EEG signals inevitably and the muscle artifacts are mainly distributed at the outer electrode sites, such as the temporary region (Muthukumaraswamy, 2013). To reduce the effect of artifacts on EEG signals, therefore, only signals acquired from FZ electrode are used to indicate the subject's neural engagement, which can be seen from Figure 3.

NeuroScan system with 256 Hz sample rate was used to acquire subjects' EEG signals. Baseline drift, which is mainly caused by spontaneous brain waves, was avoided by the removing mean method. Then the theta (3–8 Hz) and beta (12–30 Hz) bands were extracted by fast Fourier transform, and subjects' neural engagement can be calculated by Equation (10) finally.

## 3. Experiment and Results

A contrast experiment was conducted to validate the feasibility of the proposed HILO method for engagement enhancement. The experiment was approved by the ethics committee of the Institute of Automation, Chinese Academy of Sciences. All the recruited subjects were informed of the experiment contents and signed the consent forms before the experiment.

### 3.1. Experiment Design

The interactive speed-tracking riding game was used as the training task for both the control group (CG) and the experiment group (EG). More specifically, during the training, subjects should track the reference trajectory, which is constructed by four RSCs, as accurately as possible. For the CG, the proposed HILO based engagement enhancement method was not used, which was used for the EG. For the CG, the RSCs displayed on the screen were thus given randomly under the constraints of Equation (8). But for the EG, the RSCs can be optimized continuously by the HILO.

A total of 10 healthy subjects (eight men and two women aged from 24 to 29 years old), numbered from S1 to S10, were recruited to participate in the experiment. None of them knew the design process or the purpose of this study. They participated in the experiments for both CG and EG. Each experiment took about 25 min, as is similar to the commonly used period of each post-stroke rehabilitation session. The interval between the two experiments was about 20 min to give subjects enough time to rest and thus minimize the influence of the previous experiment on the next experiment results.

**Figure 4:**
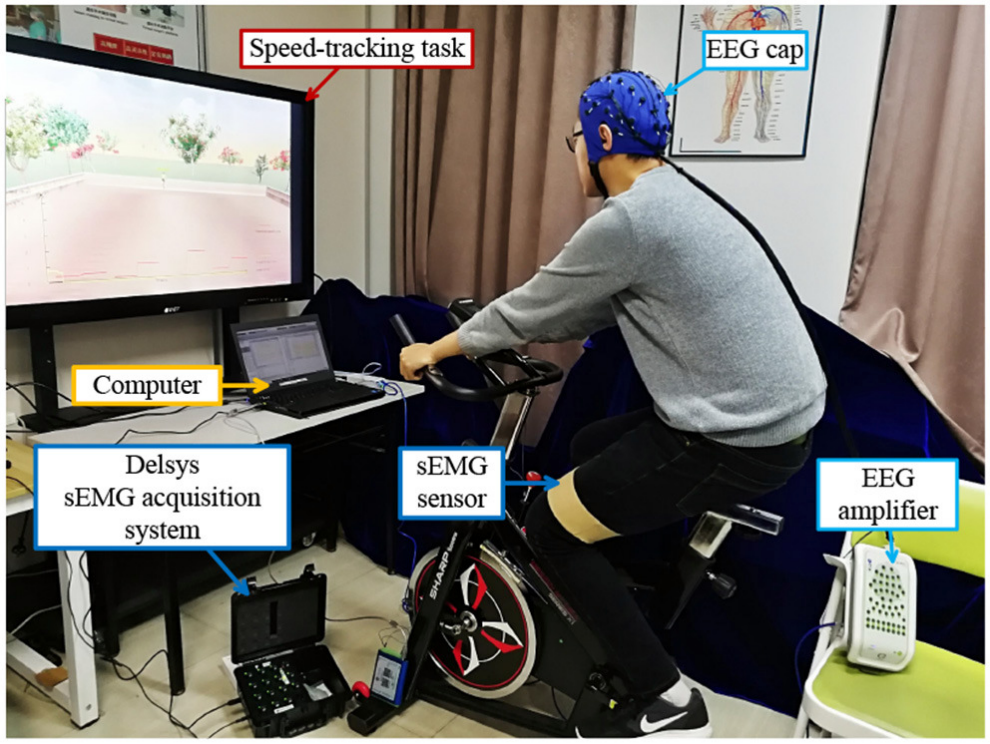
One of the experiment scenes during the training process.

During the previous 2 days before the experiment, the subjects were required to not engage in any vigorous exercises to prevent muscle fatigue and avoid affecting the collected sEMG data. To reduce possible bias, we shuffled the sequence of the experiments for the CG and EG. Subjects were able to choose which experiment to conduct first. Before the experiment, one Delysis sensor was placed on the subjects' RF muscle to acquire their sEMG signals during training, which are used to calculate their MA. Besides, an EEG cap needs to be worn to acquire subjects' EEG signals, which is used for neural engagement evaluation. All subjects received the same task instructions. They were supposed to try their best to track the reference trajectory. One of the experiment scenes during the training process is given in Figure 4. Besides, during the training, they should keep their upper body motionless to reduce muscle artifacts caused by movement.

### 3.2. Analysis of TA and MA

**Figure 5:**
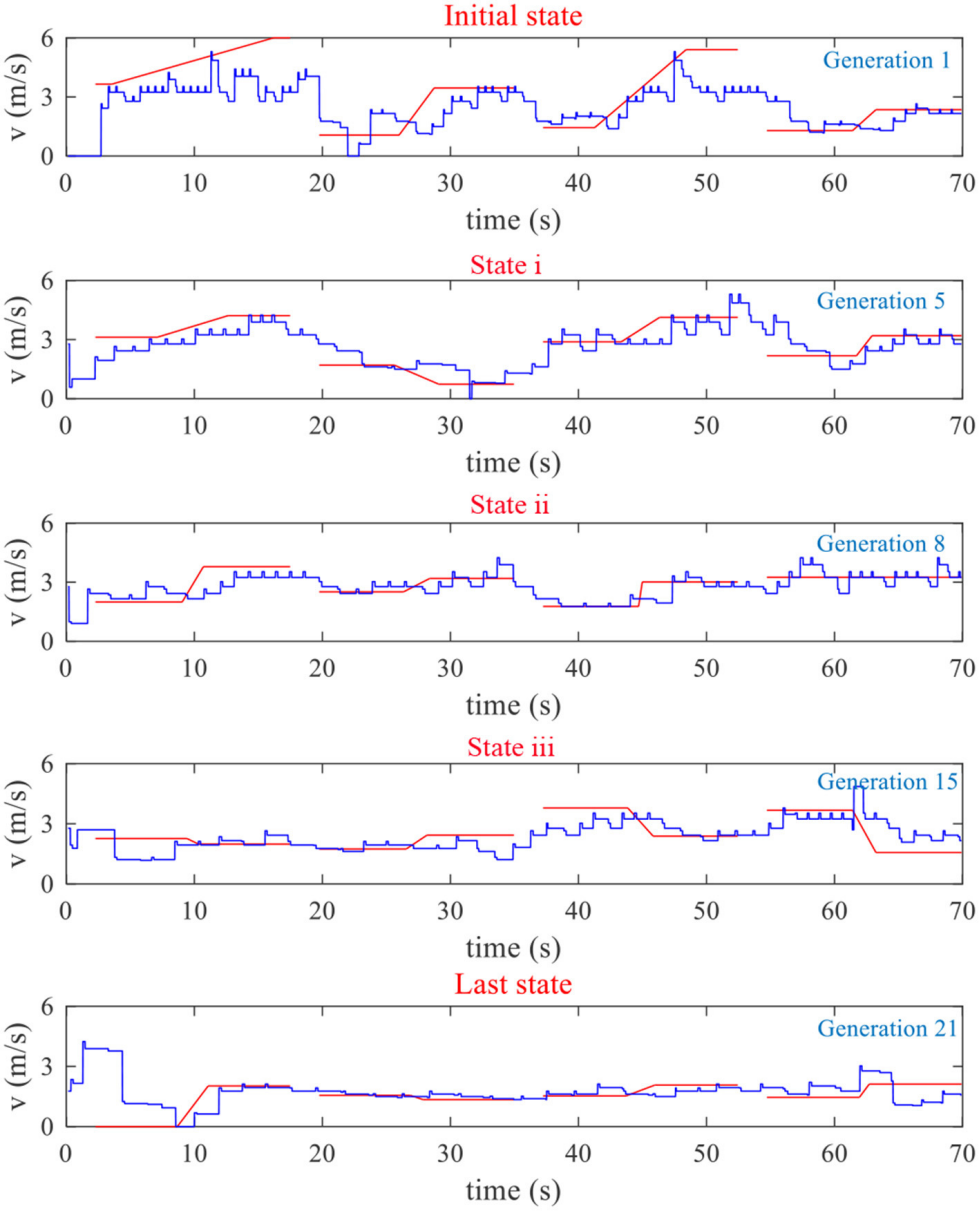
One subject's reference/actual speed curves in different generations during the training for the EG. RSCs and actual speed curves are represented by red lines and blue lines, respectively.

**Figure 6:**
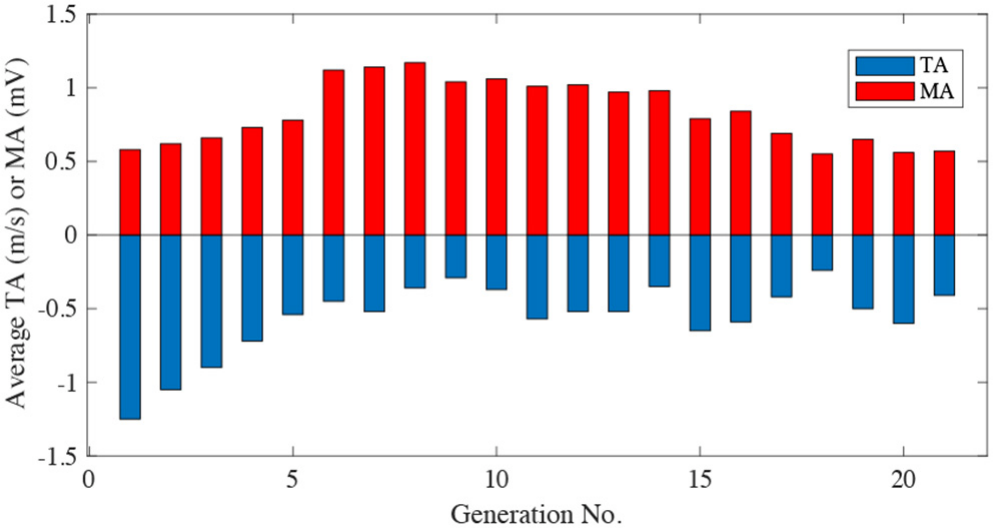
The subjects' corresponding TA and MA variations during the experiment in the EG.

For the EG, one subject's reference/actual speed curve variations are given in Figure 5, and his TA and MA during the training are given in Figure 6.

It can be seen from Figures 5, 6 that, at the beginning of the experiment, the shape of the four RSCs varied greatly, with the maximum speed reaching 6 m/s. However, the maximum cycling speed that the subject can reach was about 4 m/s. The subject could not follow the RSCs, which led to a low TA. From the initial state to state i, TA was mainly optimized to ensure that the RSCs could be tracked by the subject. The purpose of the process from state i to state ii was to improve MA as much as possible under the premise of a high TA. In generation 8, both the TA and MA were acceptable. The purpose of the process from state ii to iii was thus to maintain the subject's high TA and MA. By the 15th generation, the subject was exhausted due to a long time of training. It can be seen that from state iii to the last state, the reference speed gradually decreased to ensure that the subjects could still track the RSCs well.

**Figure 7:**
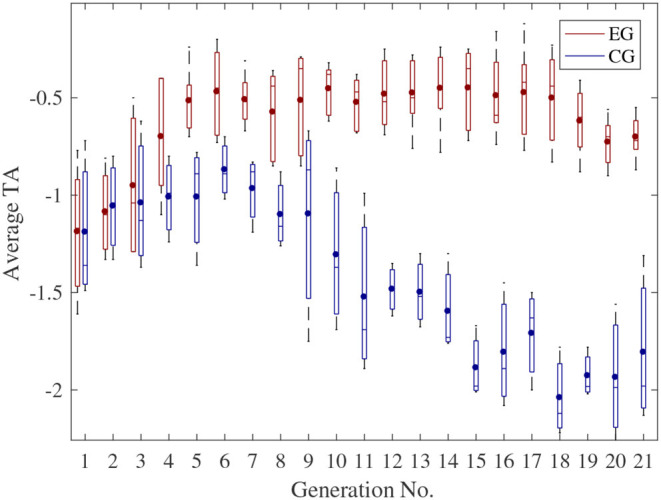
Boxplot of the average TA of all the subjects.

**Figure 8:**
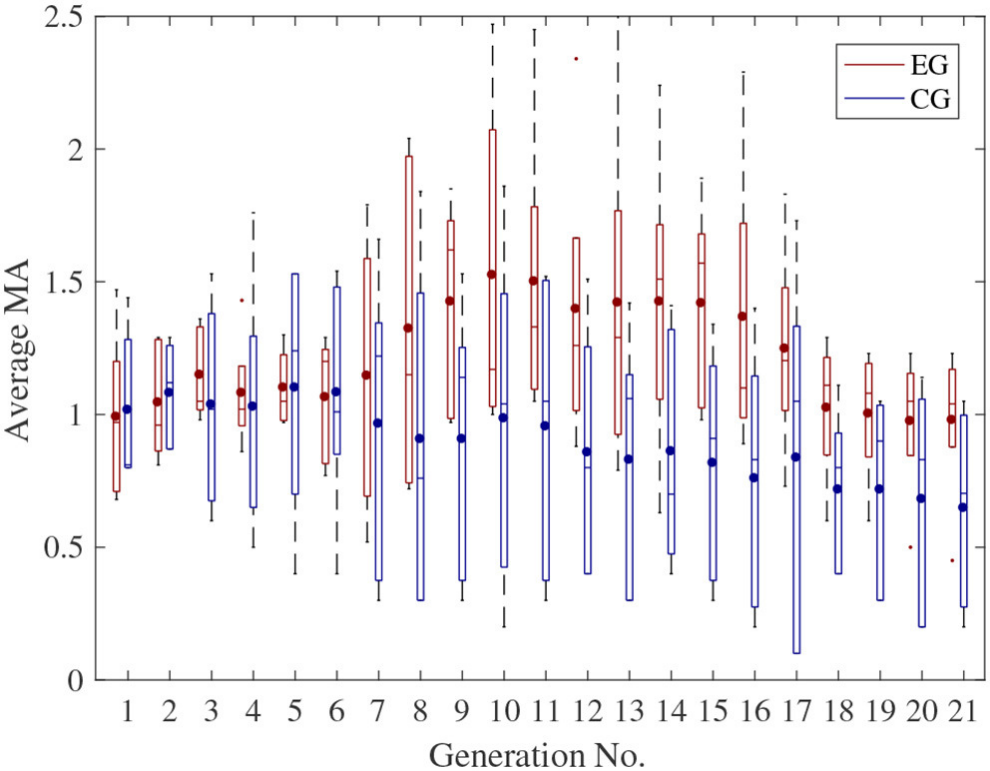
Boxplot of the average MA of all the subjects in different generations and different groups.

Boxplots of the average TA and MA of all subjects in different generations are shown in Figures 7, 8, respectively. In each box, the central line represents the median value, the dot represents the mean value, the edges of the box are the 25th and 75th percentiles. Moreover, the Wilcoxon signed-rank test results also indicate that there are significant differences between the CG and EG for both TA and MA (TA: *p*-value = 2.14e-04 < 0.0001; MA: *p*-value = 2.13e-04 < 0.0001).

It can be seen from Figure 7 that, at the beginning of the experiment, the TA for both EG and CG were relatively low since the subjects cannot track the randomly generated RSCs accurately. However, for the EG, TA can be improved obviously due to the proposed HILO. Besides, as the experiment went on, subjects became fatigued gradually, which resulted in a further decrease in the TA for the CG. This phenomenon can cause the subjects discouraged and unwilling to continue the training. However, for the EG, the difficulty of the training task can be adaptively reduced to maintain a relatively high TA. The proposed HILO method can thus result in a suitable challenging task for a specific subject to improve the enthusiasm of the subjects.

It can be seen from Figure 8 that the difference of the MA between the EG and CG was not obvious in the early stage of the experiment. One possible reason is that, in the early stage of the experiment, the main purpose of the optimization was to improve the TA due to the subjects' relatively bad tracking performance, during which the MA didn't change much for the EG.

Besides, for the CG, the ranges of both TA and MA in each generation fluctuated larger than that for the EG, especially in the later state of each experiment. One of the possible reasons is that, for the CG, the RSCs of each generation were given randomly regardless of subjects' motor ability or physiological status. The TA and MA therefore fluctuated with the variation of the given RSCs.

### 3.3. EEG-Based Neural Engagement Evaluation

**Figure 9:**
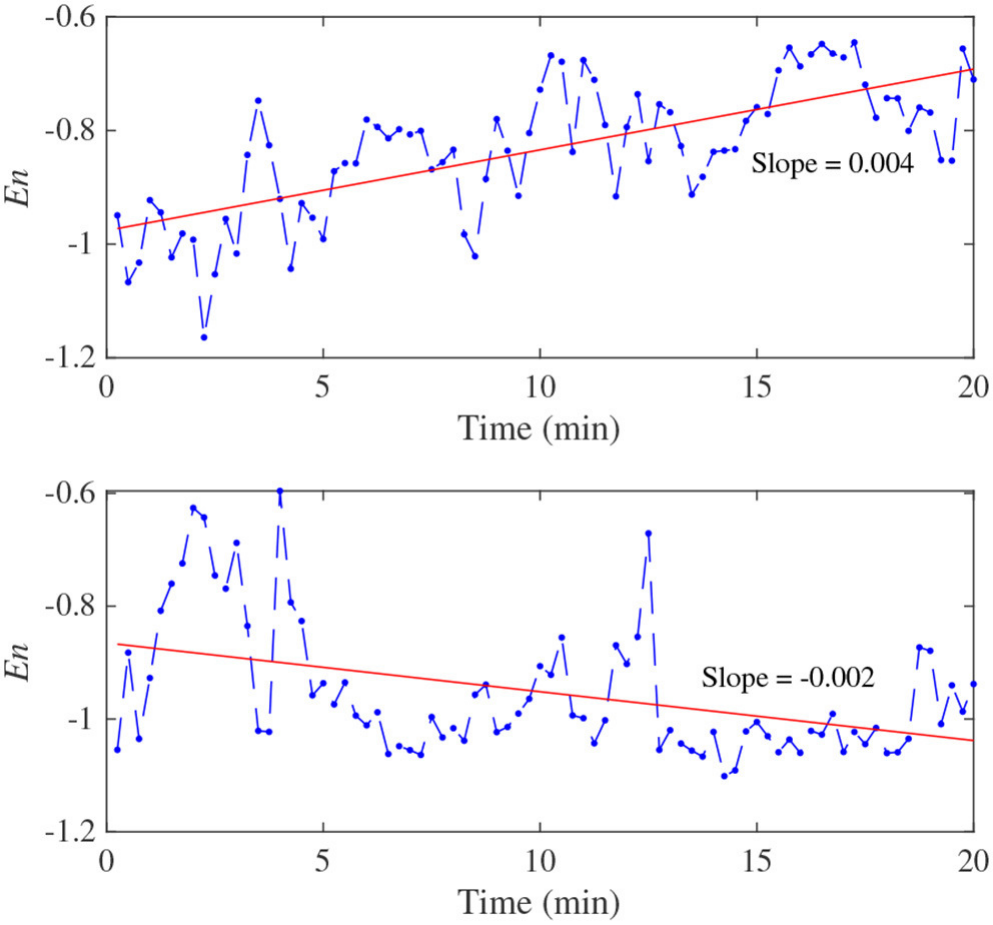
One of the subjects' EEG-based engagement variation curves and fitting curves based on a first-order linear functions. A higher *E*_*n*_ represents a higher engagement. The up and down figures are results for EG and CG, represently.

One of the subjects' EEG-based engagement variation curves and fitting curves based on a first-order linear function are given in Figure 9. The fitting curves' slopes represent the variation trends of the subject's neural engagement during training. It can be seen from Figure 9 that, for the EG, with the progress of tracking task, the values of *E*_n_ gradually increased, and these are decreased for the CG. It denotes that the neural engagement of the subject for the EG showed different degrees of improvement by using the proposed method. However, for the CG, neural engagement can be increased to some extent in the early stage (Wang et al., 2019) but dropped obviously after that.

**Figure 10:**
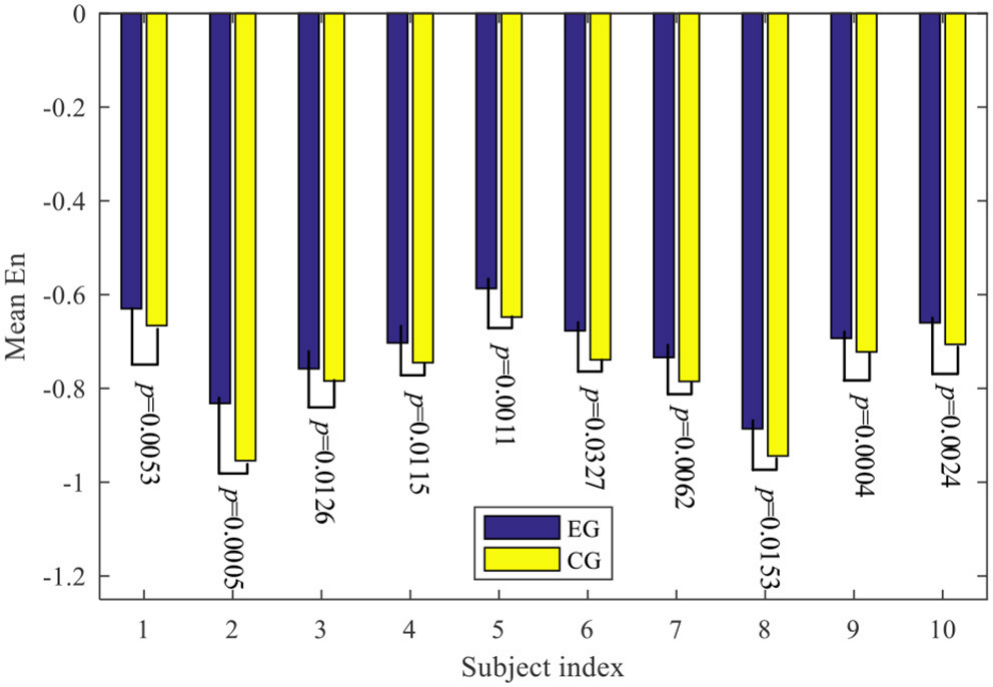
The mean values of the *E*_n_ for the 10 subjects, and the results of the significant test about the neural engagement between CG and EG.

The mean values of the *E*_n_ for the 10 subjects, and the results of the significant test about the neural engagement between CG and EG by using Wilcoxon signed-rank tests are given in Figure 10. Compared to the neural engagement in the CG, subjects' neural engagement in the EG can be improved significantly.

**Figure 11:**
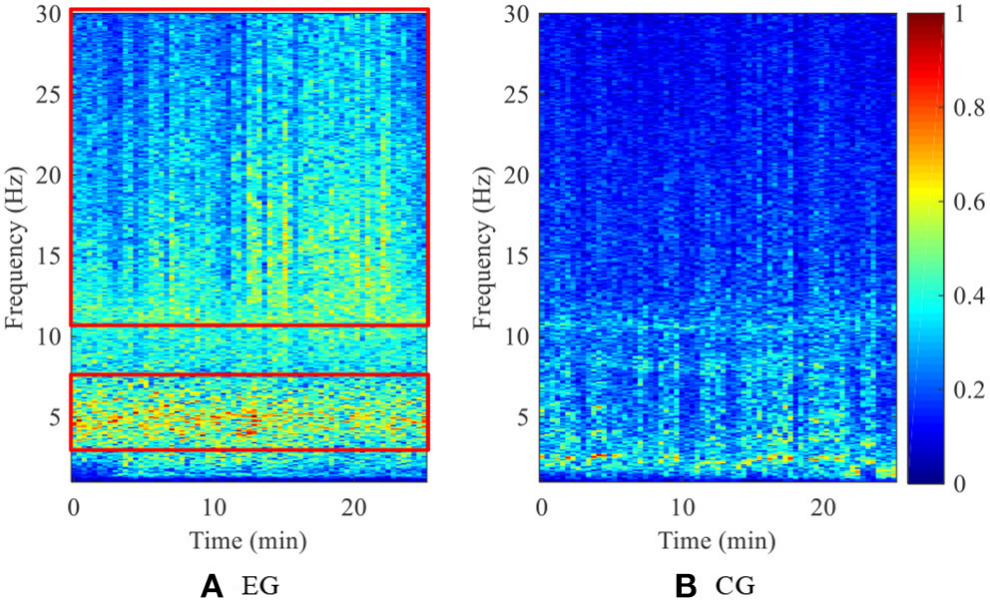
One of the subjects' time-frequency spectra during the whole training. **(A)** EG. **(B)** CG.

To clearly show the brain activity variation during the speed-tracking task, one subject's time-frequency spectra, which were obtained by short-time Fourier transformation of the EEG signals (Wang et al., 2018), are given in Figure 11. From the figure we can see that, for the EG, the energy of the beta rhythm (12–30 Hz) increased gradually, and the energy of the theta rhythm (3–8 Hz) decreased gradually after around 13 min. For the CG, there was a little fluctuation of the EEG spectrum in different frequency bands. Since good performance and high neural engagement are related to a phenomenon of decreased theta rhythm power and increased beta rhythm power, the feasibility of the proposed HILO method in engagement enhancement can be further proved by Figure 11.

## 4. Discussion

To maximize engagement during therapy and prevent frustration, it is essential to design rehabilitation exercises in such a way where they challenge patients at a difficulty level neither too simple nor too difficult (Choi et al., 2011; Metzger et al., 2014). The ability to select and maintain an engaging and challenging training difficulty level in post-stroke rehabilitation, however, remains an open challenge. In this paper, we presented an HILO based training task optimization method by which the difficulty levels of the training task can be optimized continuously to well match the subject's current motor ability and physiological state.

Several strategies have been proposed for online decision making to modify task parameters and modulate its difficulty. For example, in Metzger et al. (2014), the difficulty of the training task is adjusted based on the completion of the task to maintain the training performance of patients in a certain range. Besides, interaction forces, muscle activity, or other kinematic or physiological parameters have also been used for training challenge adaption (Krebs et al., 2003; Novak et al., 2011; Luo et al., 2019). However, due to the complexity of the training tasks and human-machine systems, the adaptive task adjustment based engagement enhancement methods can hardly find an optimal design of the training tasks, which can be found by the optimization method.

In this paper, according to subjects' current physiological state and task performances, i.e., MA and TA, the training task parameters can be optimized continuously, to make sure that the current task parameter settings are nearly optimal for engagement enhancement. The proposed optimization method can be termed as “greedy” optimization since only the subject's performance in the latest generation rather than overall superimposed performance is considered during the optimization. In this way, the system can quickly converge to the “greedy” optimal state to improve the immediate engagement. However, during the experiment, it was found that the system can fall into a local optimal situation sometimes, which should be improved in the future.

In clinical settings, selection of the training difficulty and its adaptation over the course of therapy is often determined by the experience of trained therapists and their subjective perception of a patient's abilities (Metzger et al., 2014). Our proposed method can effectively avoid the mismatch between the difficulty of the task set manually and the patients' abilities. Moreover, by considering that active engagement of the human motor and neural system is essential for functional rehabilitation, the proposed method is promising for transfer to the rehabilitation of post-stroke patients. In the future, more experiments are to be conducted to further validate the feasibility of the proposed method for enhancement of the post-stroke patients' engagement and improvement of the rehabilitation outcomes.

## 5. Conclusion

In this paper, an HILO-based engagement enhancement method is proposed to enhance subjects' engagement. Firstly, subjects are asked to track the reference trajectory, which is constructed by four RSCs, as accurately as possible. After finishing the tracking task of each generation, the value of the designed objective function, which is equal to the sum of the TA and MA, can be calculated according to subjects' responses. Then, CMA-ES is used to generate the optimal parameters of the RSCs periodically. By optimization of the reference trajectory continuously, the objective function can be maximized and subject engagement enhanced. Finally, the feasibility of the proposed HILO method in engagement enhancement is validated through the comparison experiment on 10 subjects. Experiment results show that both TA and MA can be improved significantly (*p* < 0.0001). Moreover, all the recruited subjects' EEG based neural engagement can also be improved significantly (*p* < 0.01) and maintained at a high level by using the proposed method.

## Data Availability Statement

The raw data supporting the conclusions of this article will be made available by the authors, without undue reservation.

## Ethics Statement

The experiment was approved by the ethics committee of the Institute of Automation, Chinese Academy of Sciences. The ethics approval number is IA-201947. All the recruited subjects were informed of the experiment contents and signed the consent forms before the experiment.

## Author Contributions

JW, WW, and Z-GH response for study design. JW carried out the research. JW and WW wrote part of the manuscript. JW, SR, and WS analyzed the results and prepared the figures and tables. All the authors contributed to the article and approved the submitted version.

## Conflict of Interest

The authors declare that the research was conducted in the absence of any commercial or financial relationships that could be construed as a potential conflict of interest.
